# Annotations

**Published:** 1896-02-01

**Authors:** 


### ANNOTATIONS.

### On Sudden Death.

Time after time has the country lately been shocked by news of the death of well-known public men, and while grief and sympathy are the outward manifestations of the nation's feelings, the minds of many have been stirred in other and more personal directions. It is always so. When men who are none too old drop off unexpectedly scores of others of like age ask themselves, and if they are wise they ask their doctors, whether they also have within them the seeds of maladies which may lead to like disasters. But sudden death, except from accident, is far less common than most people imagine, and death with no preceding sign of illness is very rare indeed. Practically there are but three conditions by which men going about in apparently active health are at one blow brought to the grave—heart disease, aneurism, and apoplexy. In all of these the victims may be working well up to the moment of the attack, and although only in the first two will death at all probably take place within a few minutes, even in apoplexy conscious life may be abolished almost in an instant, which to the individual is all the same. But in only a comparatively small proportion of the cases of heart disease which are met with does the danger of such a catastrophe become at all a serious one, and most of these are cases the nature and even the danger of which are fairly obvious if they are properly examined. Much, then, as the public associate sudden death with disease of the heart, such deaths, even though they may be sudden, are not usually altogether without forewarning. In apoplexy, however, matters are rather different, for although in many cases it is possible both to predicate the iminence of apoplexy, even in persons who are apparently strong and well, and by appropriate treatment to avert the catastrophe, cases do occur in which no obvious signs are present by which the danger can be foretold. These, however, are the exception, for in the majority of cases, although it may not be possible to foretell the apoplexy, it at least is possible to say that disease is present, and to give warning how to delay its progress. If every person were examined every week, although the most sudden deaths would still be those from aneurism and from heart disease, the most unexpected and unforetellable would lie in the small group of cases of hæmorrhage in the brain, arising, not from increased tension in the arteries, but from inherent weakness in the blood vessels.

### Trades Unionism in Medicine.

What do our medical brethren throughout the British Empire think of the following? "A number of medical men in a town are outweighted by one or two so-called blacklegs, and feeling injured they appeal to the General Medical Council for help. Why do they not form trades unions?" It is Dr. Samuel Wilks, writing in the *Medical Magazine*, who states the facts and puts the question; and Dr. Wilks is a member of the General Medical Council, and a man liked and trusted throughout the profession. Now, why do medical men not form trades unions, as Dr. Wilks suggests they should do? One would like the question honestly answered. Is it not because they have some antiquated and irrational notion that it would be in some way or other derogatory to their dignity? But in what conceivable way can a legitimate combination of honourable men for their self-protection be derogatory to their dignity? We would ask our medical brethren this plain question—Which is likely to do the dignity of the profession most harm, legitimate combination for honourable objects, or the advertising, the touting, and the degrading underselling which the "so-called blacklegs" of our order are indulging in with ever-increasing audacity year by year? There is only one possible answer to this question. We ask for the exercise throughout the profession of a little courage and a little common sense.

### From Butter to Brains.

It has often been argued, and not unnaturally, by theorisers, that specially prepared fatty foods should make cows yield an abnormally high percentage of butter; whilst, on the other hand, other reasoners have held that the yield of butter depends more upon the cow than upon her food. This latter view has been mostly held by practical agriculturists—the former by certain theorists. Germany, strong in both theory and practice, some little time ago put the matter to scientific proof, and demonstrated that for once the practical agriculturists had been right and the theorisers wrong. France, in her turn, has subjected the German conclusions to criticism, and to the test of further experimentation. At the experimental station at Grignon a dozen specially selected cows of various breeds have recently been experimented with in every conceivable way, and the results have in every experiment justified the German conclusion, namely, that the yield of butter depends upon the individual cow, and not in any measurable degree upon the richness, or otherwise, of her diet in fatty foods. "From butter to brains" seems a "far cry"; but it is by no means so far as it seems. Foods rich in phosphorus, such as fish and the like, have long been held by certain people to be "good for the brain." Some have even gone so far as to declare that the brains of mankind in general are capable of being permanently strengthened and clarified by a specially prepared phosphoric dietary. But it has never been scientifically demonstrated that fish and other phosphoric foods can appreciably improve the brain and mind. Fishermen, for example, and fishmongers, who may be supposed to live largely upon fish have never shown themselves to be in any measurable degree more intellectual than their neighbours. Indeed, it may be plausibly argued that they are a little less so. The truth is, that that particular food which best agrees with the particular individual, and which best maintains his general health at a high level, is the best for the brain and every other organ of the body, as well as for the whole man. Common experience has long ago formulated the saying that "what is one man's meat is another man's poison." Science now comes forward to tell us exactly the same thing, and to impress upon everyone of us the necessity of finding out the diet best suited to ourselves, and sticking to it.

